# Teen responses when a younger school-age sibling has been bullied

**DOI:** 10.1080/02673843.2014.975258

**Published:** 2014-12-10

**Authors:** Alice Sterling Honig, Nicole Zdunowski-Sjoblom

**Affiliations:** ^a^ Department of Child and family Studies, Syracuse University, Syracuse, NY, USA; ^b^ Horsham Clinic, Philadelphia, PA, USA

**Keywords:** teens responses to bullying of their young sibling, gender differences in teen advice

## Abstract

The prevalence of bullying among children, and the sometimes tragic consequences as a result, has become a major concern in schools. The larger research for this study reported on in-depth interviews with 28 elementary and middle school-age boys and girls (7–12 years) who had experienced various forms of bullying and relational aggression by their peers, mostly on school grounds, and the responses of their parents and teachers. Responses of the children's teen siblings to the younger child's revelations of being bullied are the focus of this report. In-depth interviews with each teen sibling (*n* = 28) and with each bullied child revealed how the children viewed the teen siblings' supportive strategies. Almost all the children (89%) reported that their older siblings talked with them and offered advice. The teen siblings shared with the younger ones that they too (71%) had been bullied, or they knew someone who had been bullied (18%). Teens gave the advice to ‘bully back’ to 11% and advice to ‘tell someone’ to 32% of the younger children. The children felt quite positive about their older siblings' advice (89%), which did differ depending on the bullied child's gender. Teen siblings gave advice to ‘avoid bullies’ to 77% of female and to 27% of male younger children.

## 1. Literature review

Bullying is a widespread phenomenon in schools. However, not until the early 1970s did problems associated with bullying and relational aggression and problems in dealing with bullying issues become a central subject of study in the USA (Olweus, 1993). Cyberbullying, often with anonymous smears, threats and attacks on the Internet and on mobile devices, has led to teen suicides and an increased concern for bullying problems (Smith et al., 2008; Stanglin, 2013).

### 1.1. History of bullying research

Historically, the study of bullying was restricted primarily to Scandinavian countries. Olweus (1993) initiated an early societal interest in bullying and peer victimisation under the term ‘mobbing’, when large, anonymous groups of individuals engage in harassment. In Swedish schools, Olweus (1978) gathered the first systematic data about the nature and prevalence of bullying, as reported in his book *Aggression in the Schools: Bullies and Whipping Boys*. He believed that a strong research focus could enlighten and support the work of school staff members, families, as well as scholars, and also show patterns in the lives of identified bullies and victims well into adulthood.

Public interest as well as research interest in studying bullying in the USA increased after the 1999 shootings by ‘Goth’ teens at Columbine High School in Littleton, Colorado (Garbarino & deLara, 2002; Thompson & Cohen, 2005). The Columbine tragedy was one of the first to spark a national discussion focused on the problems associated with bullying in schools. When interviewed, some girls in that school said that had they known those teens would become violent, they might not have always responded so contemptuously to the boys' greetings. Subsequent episodes of school shootings and youth suicides have added further urgency to the need to study and address bullying and more than 50 recent research references are available from the journal *Child & Youth Care Forum* (2014).

National media discussions in the USA on programmes such as CNN, NPR and even The United States White House Bullying Prevention Initiative (2014) have promoted concerns about bullying prevention. British National Development Study researchers discovered long-term negative effects for children who were bullied between the ages of 7 and 11 years. Decades later, bully victims were found to be more likely to suffer depression, anxiety and suicidal thoughts, more likely to have poorer physical health, more likely to have lower education and employment levels and less likely to have long-term partners or close friendships (Takizawa, Maughan, & Arsenault, 2014). Yet, a research focus on bullying can also provide a springboard for a sense of hope and support for those who have suffered bullying (Rigby, 2002). Despite the increased attention relating to bullying and relational aggression, important conceptual and theoretical differences about what constitutes bullying, and how best to respond, continue to exist.

### 1.2. Definitions of bullying

In the USA, bullying was originally categorised as a standard form of aggression (Griffin & Gross, 2004). Although ‘bullying’ and ‘aggression’ both have similar foundations, there are unique constructs required for each in social science research. Some researchers have identified bullying as specifically taking place over an extended period of time. Olweus' definition of bullying states, ‘a child is being bullied or victimized when he or she is exposed, repeatedly and over time, to negative actions on the part of one or more other students’ (1993, p. 9).

Some researchers believe that bullying differs from aggression in three distinctive ways. First, bullying occurs when an individual (1) specifically selects a target from the larger group, (2) then decides to victimise their target over a period time, and (3) does so in a manner that will include unique and specific physical attacks, verbal assaults and other forms of social exclusion (Espelage & Asidao, 2001; Espelage & Swearer, 2004). But even one act can be strongly felt as bullying as when a child punches another child very hard on the back in a lunch line, or a schoolmate threatens to get friends to ‘rape’ a peer. Then a single episode can surely be viewed as an act of bullying.

In recent decades, scholars have learned more about the complex nature of bullying and *relational aggression* (Bowes, Maughan, Caspi, Moffitt, & Arseneault, 2010; Garbarino & deLara, 2002; Sawyer, Mishna, Pepler, & Wiener, 2011; Smith, Dowie, Olafsson, & Leifooghe, 2002). Negative actions are further identified as ‘someone intentionally inflicting injury or discomfort on another individual’ (Olweus, 1993, p. 9). Aggressive behaviours can be physical, such as beating, kicking, face-slapping or punching. But negative actions can occur without physical interaction, as when one youngster gestures contemptuously at another, makes a sneering remark or offensive facial expression, intentionally and ostentatiously excludes a peer from a group or deliberately spreads malicious rumours or gossip, purposefully and blatantly betrays confidential information from a peer, or uses verbal, racial or religious slurs and jeering name calling. ‘Cyberbullying’ where anonymous electronic bullying messages are posted online has devastated some victims.

Some researchers have disagreed with Olweus' specification that ‘intention’ and ‘repetition’ are necessary for characterising bullying. deLara (2012) has noted that Olweus' definition may disempower children and adolescents from reporting acts of bullying from their own perspective. For example, a child might well view a peer's ‘being mean’ as an act of bullying and feel helpless. Smith and Sharp (1994) agree and note that

a student is being bullied or picked on when another student says nasty and unpleasant things to him or her. It is also bullying when a student is hit, kicked, threatened, locked inside a room, sent nasty notes, and when no one ever talks to him. (p. 1)

This definition of bullying does not require event repetition.

Thus, ‘children conceive of bullying in broader terms without necessarily invoking intention, repetition of actions, and power imbalance between aggressors and victims’ (Kanetsuna, Smith, & Mortia, 2006, p. 570). Adults need to ask children themselves to tell when they consider an action as mean or unkind bullying. The consequences of *relentless bullying* can be severe and tragic and result in child suicide as in the September 2013 case history below, which should serve as a ‘wake-up call’ for families to monitor children's use of use of internet sites.

The sheriff who arrested two girls for allegedly bullying a 12-year-old into committing suicide says authorities are trying to decide whether they can also charge one of the suspect's parents. The pair – ages 12 and 14 – were arrested on Monday and charged with third-degree felony aggravated stalking in connection with the death of S. Rebecca of Lakeland, Florida, who jumped from a cement factory tower on 9 September. Rebecca, who, authorities say, was bullied relentlessly for months, was ‘terrorised’ online. One message to Rebecca said she should ‘drink bleach and die’. Polk County Sheriff Grady Judd said that the bullying began after the 14-year-old suspect began dating a boy Rebecca had been seeing. She ‘began to harass and ultimately torment Rebecca’, Judd said, and prodded the 12-year-old to join in. ‘I'm aggravated that the parents aren't doing what parents should do … Responsible parents take disciplinary action’. Judd told NBC's *Today*. He said the parents of the 14-year-old suspect are in ‘total denial’. ‘They don't think there is a problem here, and that is the problem’; he added that the girl's parents gave her back her Facebook access even after learning about her alleged bullying of Rebecca. ‘That's terrible’, he said. ‘That's why we moved fast to lock their daughter up’. The sheriff said the tipping point leading to the arrests came when the older suspect allegedly posted on Facebook: ‘Yes I know I bullied Rebecca and she killed herself but IDGAF [I don't give a (expletive)]’. ‘We decided, look, we can't leave her out there’, the sheriff told reporters. ‘Who else is she going to torment? Who else is she going to harass? Who is the next person she verbally and mentally abuses and attacks?’ The suspect told deputies that her Facebook account was hacked and that she did not write that post, WTSP-TV reported. Sheriff Judd, noting the suspects have clean criminal records, said that the girls – if convicted – are not likely to serve much time in jail, if any (Lakeland Girl Commits Suicide, 2013).

### 1.3. Social group mechanisms

Important social group mechanisms often exist in a bullying situation. In a study of *school climate for bullying*, from anonymous questionnaires given to 2240 middle and high school students, Nickerson, Singleton, Schurz, and Collen (2014) reported that 362 male and 421 females had been bullied, but *fewer than half had reported the bullying*. More than 1500 youths reported *witnessing* bullying. About 400 reported bullying others, and more than 1300 reported having tried to help others who were bullied. Thus, bullying in schools is a widespread and serious problem.

Group processes have been discussed in terms of (1) a social contagion, (2) the weakening of control or inhibitions against aggressive tendencies, (3) diffusion of responsibility, and (4) and the gradual cognitive changes in the perception of bullying and of the victim. *Social contagion* occurs when group members follow a model of aggression provided by the bully. The second dimension refers to *bystanders* who are slow to take initiative, but are willing to participate in bullying when someone else has started it. *Diffusion of responsibility* occurs when multiple players take part in bullying, and feelings of guilt diminish. Lastly, *cognitive changes* occur when classmates start to perceive the victim as increasingly deviant with time, believing that he or she is deserving of punishment (Juvonen & Graham, 2001; Salmivalli, 2001).

### 1.4. Sibling relationships

The social support literature reflects both positive and negative positive aspects of sibling relationships. Sibling relationships can involve levels of trust and camaraderie that can be both helpful and supportive to a child and also enhance a young child's growth in prosocial behaviours such as turn taking, helping and sharing (Stormshak, Bellanti, & Bierman, 1996). Younger children can learn social and behavioural tactics from older siblings to help them handle difficult events such as being bullied. Children who have affectionate relationships with their siblings are less likely to develop emotional problems over time when compared to children who do not have affectionate relationships with their siblings (Bowes et al., 2010). Sibling relationships can be among the most important in a person's life, largely due to their levels of intimacy, familiarity and emotional strength (Bank & Kahn, 1997). Older siblings can provide a long-term and steady sense of companionship, acceptance and warmth for younger children in early and middle childhood (Dunn & Kendrick, 1982).

Some interactions between siblings may lead to negative outcomes. Siblings sometimes trigger aggressive behaviours in one another, such as fighting and name-calling (Menesini, Camodeca, & Nocentini, 2010; Patterson, 1986). This can be of particular importance in a bullying situation where a child might learn violence or aggression in the home (Evans, Garner, & Honig, 2014). Clinicians have revealed negative long-term consequences (mental health difficulties, violent behaviour) among children who have witnessed, or experienced abuse and violence in a domestic situation (Osofsky & Fenichel, 1996). In a Colorado study of how siblings felt about each other, Dunn and Plomin (1990) cite parent reports that ‘60% of the children differed from their sibling in the extent of their positive and friendly feelings and behaviour toward that sibling’ (p. 105).

Thus, in the current study we wished to discover not only how teens felt and behaved when learning about a younger child having been bullied, but also how they responded and how the bullied child felt about the teen's responses to their disclosure.

### 1.5. Theoretical frameworks

Stage theories of development using the conceptual frameworks of Jean Piaget, Erik Erikson, as well as Albert Bandura's social learning theory are important for understanding the behaviours and feelings of teens and their younger bullied siblings. According to Piaget (1965), children in the concrete operational stage of development (7–11 years) become increasingly able to shift away from a self-centred perspective to a viewpoint that allows for more mature moral judgements and consideration for the viewpoints of others. Thus, positive experiences during this stage are critical for a child's psychological and emotional development in relation to healthy peer group interactions. Rejection and isolation from one's peers can be detrimental to a child's general psychological development and have been linked with lowered self-esteem, and delayed social learning (Murray-Close, Ostrov, & Crick, 2007; Ostrov, Crick, & Stauffacher, 2006; Rodkin, 2003).

Erikson (1968) theorised that children between 6 and 12 years of age developmentally undergo a crisis he labels ‘industry vs. inferiority’, such that children may be at risk for developing feelings of inadequacy when teased or rejected by their peers. When bullied, children during this stage may also be at risk later on for developing problems of lower social competence and self-esteem, rather than mastery of important social and emotional skills including *self-regulation*, that will help them adapt to a larger social environment. When bullied, children receive positive support, such as reassurance from an older sibling; they may then be better equipped to deal with difficult peer challenges.

Bandura's (1973) social learning theory provides another important lens to regard bullying and relational aggression. Social learning theory emphasises concepts such as modelling and imitation of behaviours from social cues in the environment. Children learn aggression behaviours as they engage in various forms of modelling and imitation of family members, as well as individuals in their peer group. In a situation where a younger child is bullied, he or she may be at the mercy of a bully who learned aggressive and abusive behaviours from interactions in the home. A child might also learn methods of dealing with a bully from behaviours seen at home, such as hitting back or trading curses.

Overall atmosphere in the home may serve as a supportive buffer to help a young child deal with the negative effects of bullying and relational aggression (Bowes et al., 2010). Using Bronfenbrenner's (1986) model of social-ecological contexts including the *micro* (family), *meso* (family–school) and *macro* (religion/culture, political) spheres to study bullying prevention and intervention in South Korea, Hong, Lee, Lee, Lee, and Garbarino (2014) have shown a *positive moderating effect of family nurturance* on the chances that a fearful/anxious child would be bullied.

### 1.6. Research goals

The research reported here uses deLara's (2012) definition of bullying, which states that bullying occurs ‘when someone is being mean’, and may also involve other forms of direct and indirect harassment. This study focuses on older teen sibling's responses to a younger child who has been bullied. The children themselves were encouraged to self-disclose acts of ‘meanness’ from their own perspective as acts of ‘bullying’. The responses of the bullied children and of their parents have been presented elsewhere (Honig & Zdunowski-Sjoblom, 2014). Because family relationships, and particularly those with older siblings, can provide insights for ameliorating the negative effects of bullying, this report focuses on child feelings about confiding bullying *experiences* to an older sibling, *how* that adolescent sibling responded to the news about the bullying and what the bullied children's feelings were in response to their teen siblings' advice.

## 2. Method

Interviews were conducted individually in middle-income families, with 28 adolescents, 13–17 years of age, and with their younger siblings, 7–12 years of age, who had experienced various forms of bullying and relationship aggression by their peers. The majority (24/28) of families were White (see Tables 1 and 2).

**Table 1:** Parent demographic information.

*n* = 28	*M*	Median	Range
Parent age	40.79 (4.09)	41.00	34–48
Female (*n* = 27)	1.0	1.0	
Male (*n* = 1)	2.0	2.0	
Parent income			
1 = $30,000 or less (*n* = 3)			
2 = $30,001–$60,000 (*n* = 14)	2.29	2.00	$30,001–$60,000
3 = $60,001 and above (*n* = 11)	(0.659)		
Parent education			
Graduate degree (PhD, MA, MS)	*n* = 5		
College degree (BS, BA, AS)	*n* = 18		
High school degree or less	*n* = 5		
Marital status			
Single	*n* = 5		
Married, living with partner	*n* = 16		
Unmarried, living with partner	*n* = 5		
Divorced	*n* = 2		

Note: Standard deviation is located in parenthesis below the mean.

**Table 2:** Child age, gender, and sibling age separation.

*n* = 56	*M*	Median	Range	
Older siblings	14.11 (1.31)	14.00	13–17 years	
Older/male (*n* = 14)	14.36 (1.55)	14.00	13–17 years	
Older/female (*n* = 14)	13.86 (1.02)	13.50	13–16 years	
Younger siblings	9.89 (1.98)	10.50	7–12 years	
Younger/male (*n* = 15)	9.60 (1.99)	10.00	7–12 years	
Younger/female (*n* = 13)	10.23 (2.00)	11.00	7–12 years	
*n* = 28 pairs	Older teen gender	*M* age separation	Younger age gender	Age separation sibling (range)
*n* = 7	OM	3.85 (1.21)	YM	2–5 years
*n* = 7	OM	3.85 (1.57)	YF	2–6 years
*n* = 8	OF	5.25 (2.54)	YM	2–8 years
*n* = 6	OF	3.66 (1.50)	YF	2–6 years

Note: Standard deviation is located in parenthesis below the mean. OM, male sibling; YM, younger male child.

### 2.1. Procedures

All research materials and interview questions were reviewed, approved and renewed on a yearly basis by the University Institutional Review Board (IRB). After receiving IRB permission, advertisements were given out at child and family health centres, libraries, websites, bulletin boards, supermarkets, shopping malls and elementary schools in three states in the north-eastern USA (New York, Maryland and Pennsylvania). All interviews were carried out individually in the home. The identified categories of bullying experiences were then coded by two Child Development graduate students, blind to the study objectives, who achieved a Cohen's kappa = 0.725 for inter-rater agreement.

### 2.2. Measures

Each teen and each bullied child responded to a series of open-ended questions regarding the child's experiences with bullying and relational aggression. School grounds were the predominant locale of the reported bullying for 85% of female children and for 67% of the male children. The bullied children shared what they had told their teen siblings and how they felt about that experience and any advice offered. The teens explained what they said and did and their personal feelings about the younger child's bullying situation.

To begin, the interviewer (NZ) read a short children's story about childhood bullying to each younger child and then asked a series of open-ended questions about the child's own personal experiences. The 26-questions interview addressed (1) examples of bullying that took place, (2) thoughts and feelings about being bullied, (3) the child's reaction to being bullied, (4) help sought from an older brother or sister, (5) feelings about the older sibling's response to the situation, and (6) other possible sources of support available to the child.

### 2.3. Teen sibling questionnaire

Teen siblings were asked 15 open-ended questions in order to learn their perspectives on the younger child's bullying situation. The questions addressed (1) being approached directly by the younger child about being bullied, (2) information the younger child gave about the type of bullying experienced, (3) the emotional reaction of the teen sibling to the child's situation, and (4) types of support (if any) provided by the teen to the younger sibling. Supports could include actions such as listening, talking with the target child, offering advice, sharing stories or telling an adult or teacher.

#### 2.3.1. Data analysis

Chi Square and Fisher's Exact Tests, as well as descriptive statistics, were used to analyse data for children and their teen siblings.

## 3. Results

### 3.1. How likely was a child to disclose the bullying to an older sibling?

Regardless of whether the older teen was a brother or sister, overwhelmingly teens reported that the younger child did tell them about their bullying experiences. That is, 100% of female teens and 93% of male teens reported that the younger sibling approached them about being bullied. Teen gender did not affect the willingness of the bullied child to discuss their bullying experience (χ^2^ (2, *n* = 28) = 1.00, *p* = 0.60). Given that overwhelmingly, the younger children did share with their teen siblings the kinds of bullying they had experienced, it is of interest to note what *kinds* of harassment children reported depending on whether the teen was female or male.

Teens identified several types of bullying experienced by their brothers and sisters: 36% of females reported that their brother or sister experienced verbal harassment, 36% reported that their sibling experienced relational aggression and 28% shared that their sibling experienced physical harassment. Among male teens, 50% reported that their sibling experienced verbal harassment, 36% shared that their sibling experienced relational aggression and 14% reported that the younger child had been physically harassed (see Table 3).

**Table 3:** Teen reports of types of bullying the younger sibling had experienced.

What the children shared about their bullying experiences	Gender of teen sibling		Total
	Female	Male	
Experienced verbal harassment	5 (36)	7 (50)	12 (43)
Experienced relational aggression	5 (36)	5 (36)	10 (36)
Experienced physical harassment	4 (28)	2 (14)	6 (21)
Total	14 (100)	14 (100)	28 (100)

Note: Percentages in parenthesis each number.

For the total group, 43% of teens reported that their younger brother or sister experienced verbal harassment, 36% reported that their sibling experienced relational aggression and 21% reported that their sibling was bullied physically. No gender differences were found in the percentage of children reporting which kinds of bullying had occurred, regardless of whether the teen was male or female (Fisher's Exact Test, *p* = 0.54).

### 3.2. What did the bullied children themselves report about the types of bullying experienced?

The younger children were asked to identify their most recent types of bullying experiences. Among females, 15% reported verbal aggression, 77% reported relational aggression and 8% reported physical aggression. Among males, 67% reported verbal aggression, none reported relational aggression and 33% reported physical aggression. For the total group of males and females, 43% reported verbal aggression, 36% reported relational aggression and 21% reported physical aggression (see Table 4). There were *significant gender differences in the types of bullying experienced by the children* (χ^2^ (2, *n* = 28) = 17.9, *p* = 0.00). Girls experienced more relational aggression than boys, and boys experienced more physical and verbal aggression than girls.

**Table 4:** Types of bullying the younger children themselves reported.

Type of bullying	Gender of target child		Total
	Female	Male	
Verbal aggression	2 (15)	10 (67)	12 (43)
Relational aggression	10 (77)	0 (0)	10 (36)
Physical aggression	1 (8)	5 (33)	6 (21)
Total	13 (100)	15 (100)	28 (100)

Note: Percentage in parenthesis below each number.

### 3.3. Teens' feelings on learning about the younger child's bullying experience

The teen siblings were asked to share how they felt when they learned the younger child had been bullied. *Overwhelmingly, the teens reported feeling distressed* when they learned about the bullying (sadness, anger, frustration). Among female teens, 86% reported a negative emotional response to the news of the bullying, while 14% reported feelings of indifference to the situation. Hundred per cent of male teens reported feeling a negative emotional response (sad, angry and upset) about the younger child's bullying situation.

For the total group of adolescents, 93% of teens reported experiencing a negative emotional response, and 7% reported feeling indifferent. There were no significant gender differences in the teens' emotional reaction to news of the child's bullying experiences (Fisher's Exact Test, *p* = 0.241).

### 3.4. Bullied children's discussion of the responsiveness of the teen sibling

Bullied children were asked about ways in which their older sibling had responded to the news of their bullying. All the female younger children (100%) reported speaking with teen sibling about their bullying experiences, while 8% reported that their sibling spoke with someone else on her behalf. A majority of bullied children (92%) reported that their teen sibling talked with them and also offered advice. There were no significant gender differences in the younger children's responses about talking with their teen siblings (χ^2^ (2, *n* = 28) = 0.90, *p* = 0.64).

Among males, only one child reported not speaking with the older sibling, 7% reported the sibling spoke with someone else on their behalf (a parent or teacher) and 87% of bullied males (87%) reported that their sibling did talk with them. The majority of the bullied children (89%) reported that their older sibling did talk with them and also offered advice and two children said the teen sibling then spoke with someone else on their behalf (see Table 5).

**Table 5:** Child's report about the teen sibling's verbal responses on hearing of the bullying.

Teen sibling's response to the bullied child	Gender of bullied child		Total
	Female	Male	
Did not talk with teen	0 (0)	1 (7)	1 (4)
Teen spoke with someone else on my behalf	1 (8)	1 (7)	2 (7)
Teen talked with me, offered advice	12 (92)	13 (87)	25 (89)
Total	13 (100)	15 (100)	28 (100)

Note: Percentages in parenthesis below each number.

### 3.5. Teens shared personal experiences with the bullied younger sibling

Teens reported sharing personal bullying experiences with their bullied younger siblings. Among female teens, 64% shared that they had also experienced bullying, 29% shared that they knew someone who had been bullied and 7% did not speak with their sibling about their own experiences. Of the male teens, 79% shared that they had also been bullied, 7% shared that they knew someone who had been bullied and 14% of males did not speak with their siblings about their own experiences (see Table 6).

**Table 6:** Personal shared responses of teen siblings on hearing of the bullying situation.

Teen sibling verbal response	Gender of bullied child		Total
	Female	Male	
I also was bullied	11 (85)	9 (60)	20 (71)
I know someone who was bullied	1 (8)	4 (27)	5 (18)
Teen did not share own experiences	1 (8)	2 (13)	3 (11)
Total	13 (100)	15 (100)	28 (100)

Note: Percentages in parenthesis below each number.

For the total group, *the majority of teens (71%) revealed that they had also experienced bullying*, 18% reported that they knew someone else who had been bullied and 11% reported not speaking with their sibling about their personal experiences (see Table 6). There were no significant gender differences in the types of disclosure provided to the younger child by his or her sibling (χ^2^ (2, *n* = 28) = 2.33, *p* = 0.31).

### 3.6. What the bullied children reported about their older sibling' self-disclosures upon hearing about the bullying

The majority of the bullied children *confirmed* that their older teen sibling had revealed that they too, or someone they knew, had also been bullied in the past. Of the bullied females, 85% reported that their siblings shared that they had also experienced bullying. One female child reported that her teen sibling knew someone who had been bullied, and another female reported that her sibling did not share experiences.

Among the younger males, 60% reported that their teen sibling shared that they had also been bullied, 27% reported that their sibling knew someone who was bullied and 13% reported that their teen sibling did not share personal information.

For the total group, 71% of bullied children reported that their teen sibling shared that they had also been bullied, 18% had a sibling who knew someone that had been bullied and 11% reported that their sibling did not share about their own experiences. There were no significant gender differences among the younger children in types of teen personal responses they reported (χ^2^ (2, *n* = 28) = 2.20, *p* = 0.33).

### 3.7. Teens' feelings about communicating with the child about their own bullying experiences

Teens were asked to discuss their own feelings regarding communication with the younger child about their own bullying experiences. Among female teens, 93% reported experiencing a positive emotional response to their personal disclosure about also being bullied. Seven per cent of female siblings reported not speaking with their brother or sister about bullying. Of male teens, 86% reported experiencing a positive emotional feeling having shared their personal experience, while 14% of children reported not speaking with their bullied younger sibling

For the total group, *89% of the teens reported having felt a positive emotional response to their personal disclosure about bullying*, and 11% reported not speaking with the younger child (see Table 7). There were no significant gender differences in sibling feelings about personal disclosure (Fisher's Exact Test, *p* = 0.50).

**Table 7:** Teen siblings' feelings about personal disclosure.

Teen feelings about personal disclosure	Gender of teen		Total
	Female	Male	
Positive emotional response	13 (93)	12 (86)	25 (89)
Did not speak with younger sibling about bullying, or relate to their situation	1 (7)	2 (14)	3 (11)
Total	14 (100)	14 (100)	28 (100)

Note: Percentages in parenthesis below each number.

### 3.8. What kinds of advice did children report that teen siblings provided?

*Advice was a prominent feature of teen responses* on hearing that their younger sibling had been bullied. All the bullied younger female children (100%) and a majority of males (87%) reported that their older sibling offered advice about how to handle their bullying experiences – 93% of the total group. Two males (13%) reported receiving no sibling advice.

#### 3.8.1. Teen advice varied by younger child's gender

The type of advice that older siblings offered to the bullied child seemed to depend on the younger child's gender. Among bullied females, 15% reported that a sibling told them to tell someone about being bullied, while 77% reported that a sibling told them to avoid their bullies altogether and 8% reported that a sibling told them to bully back.

For males, 47% reported a teen sibling told them to tell someone, 27% were told to avoid the bullies entirely, 13% reported that their teen sibling told them to bully back and 13% had siblings who did not offer any kind of advice.

For the total group, 32% reported that their teen sibling told them to tell someone, while half (50%) were told to avoid the bullies altogether; 11% of children were told to bully back, and 7% reported no advice offered (see Table 8). Thus, there was a trend towards significance, so that the *type of advice offered depended on whether the bullied younger child was male or female*. (χ^2^ (3, *n* = 28) = 7.58, *p* = 0.056).

**Table 8:** Type of advice the bullied child reported that the teen sibling provided.

Type of advice provided by teen sibling	Gender of bullied child		Total
	Female	Male	
Tell someone	2 (15)	7 (47)	9 (32)
Avoid bullies	10 (77)	4 (27)	14 (50)
Bully back	1 (8)	2 (13)	3 (11)
Teen did not offer advice	0 (0)	2 (13)	2 (7)
Total	13 (100)	15 (100)	28 (100)

Note: Percentages in parenthesis below each number.

### 3.9. Bullied children's emotional reaction to siblings' advice about the bullying situation

Overwhelmingly, *both boys and girls reported* *positive feelings* about their older siblings talking with them about their bullying situation. The majority of females (92%) reported a positive feeling, and one younger female reported a negative feeling. Among the males, 87% reported feeling positive, one younger male reported feeling negative and one male did not speak with his teen sibling about being bullied.

For the total group, 89% reported a positive feeling, and 7% reported a negative feeling. There were no significant gender differences in the children's feelings about their siblings' response to their bullying situation (see Table 9). The majority of children reported a positive feeling about their teen sibling's response (χ^2^ (2, *n* = 28) = 0.902, *p* = 0.064).

**Table 9:** Children's feelings about teen siblings' response to the bullying situation.

Child's emotional reaction to teen sibling response	Gender of bullied child		Total
	Female	Male	
Positive feeling	12 (92)	13 (87)	25 (89)
Negative feeling	1 (8)	1 (7)	2 (7)
Did not talk with sibling	0 (0)	1 (7)	1 (4)
Total	13 (100)	15 (100)	28 (100)

Note: Percentages in parenthesis below each number.

### 3.10. Teen reports on telling a parent about the younger child's bullying situation

The teens were asked to reflect on and discuss whether they had ever approached a parent about their brother or sister's bullying troubles. Half of females (50%) reported telling a parent, and the other half (50%) reported not telling a parent about the target child's experiences. For males, 7% reported telling a parent about the bullying. *The majority (93%) of teen males said that they did not tell a parent about the younger child having been bullied.*

For the total group, 29% of teens told a parent, and 71% reported not telling a parent (see Table 10). There were significant gender differences in male and female teen reports of telling a parent about the younger child's bullying troubles. Females were equally willing and unwilling to tell a parent about their sibling's experiences, but *male teens were far less likely to tell a parent* (Fisher's Exact Test, *p* = 0.02).

**Table 10:** Teen told a parent about the younger child's bullying situation.

	Gender of teen		Total
	Female	Male	
Yes	7 (50)	1 (7)	8 (29)
No	7 (50)	13 (93)	20 (71)
Total	14 (100)	14 (100)	28 (100)

Note: Percentages in parenthesis below each number.

Those eight teens who had disclosed to a parent were asked how they felt about telling a parent about the younger child's bullying troubles. Their responses were predominantly negative. Among the seven females, only one reported a positive emotional response and six reported a negative emotional response. The one male teen who did tell a parent reported feeling negative about disclosing. For the total of eight teens interviewed who had told a parent, only one felt positive; the other 20 teens did not share the younger child's bullying troubles with a parent.

## 4. Discussion and conclusions

In this research, individual interviews with teen siblings and with their younger bullied sisters and brothers provided increased insight into the prevalence of a variety of types of bullying that school children experience. The research also offered insights into the kinds of bullying that school-age children confided to their older brothers and sisters. Verbal harassment and relational aggression were reported more frequently than physical aggression. Regardless of type of bullying experienced, all the younger siblings reported quite negative feelings. Thus, when adults tell children: ‘Forget it. Kids will be kids’, this adult response does not address honestly, nor does it heal the negative feelings of the bullied children, nor does it provide the bullied children with coping skills for dealing with bullying peers.

### 4.1. Emotional hurts are perceived as bullying

The research findings suggest that adults need to become more aware of how hurtful non-physical bullying experiences can be in their impact on school children. The old adage – ‘Sticks and stones may break my bones but words can never harm me’ – was not true for any of the children interviewed for this research.

There were significant *gender differences* in reports of the three types of bullying. Girls reported more relational aggression than boys, and boys reported more physical and verbal aggression experiences compared with girls. Adults need to be aware of and find ways to address all types of bullying that children report, including verbal and relational bullying, as well as physical menacing and aggressions.

### 4.2. Teen siblings: a positive support system for bullied younger children

The children who disclosed their bullying situations reported overwhelmingly that they felt quite positive about their disclosures to their teen siblings. The children were also quite clear in reporting that their older brothers and sisters tried to be helpful when approached about the bullying. Most of the bullied children (89%) reported that their teenage siblings provided advice when the children communicated the circumstances of having been bullied. These data about teen responses to revelations about the bullying episodes increase our awareness that one positive help for bullied children could be to enlist the support of their teenage siblings or perhaps the support of close teenage cousins.

One of the empathetic responses of the teens was to reveal to their younger siblings that they too (71%) had experienced bullying. About one-fifth of the teens reported that they knew someone who had been bullied. Thus, older siblings of bullied children in this study were willing to self-disclose and be helpful in talking with their younger siblings who had been bullied. Of the total group of teens, 89% *reported feeling positive about personal disclosure in their talks with the younger bullied sibling*. Enlisting adolescents to be helpful when younger siblings have been bullied thus can sometimes provide an opportunity for enhancing sibling closeness and also increase teen feelings of ease in self-disclosure to a younger brother or sister who has experienced bullying troubles.

The children reported that most of their teen siblings (about 90%), *regardless of child gender*, provided advice for them. However, gender differences were clear in the *specific* teen responses, which depended on the gender of the younger child. When the bullied child was a female, then teen siblings (77%) urged the child to ‘avoid bullies’. Only 27% of teens with male younger siblings gave the same advice. Unfortunately, children cannot always avoid a bully. So this advice may not help a child avoid another episode, as bullies are sometimes persistent in their harassment. Nearly half of the teens with male younger siblings urged the children to ‘tell someone’. However, if teachers are likely to shrug off non-physical bullying as if that is ‘just how kids are’ such counsel, as Garbarino and deLara (2002) have reported from high school interviews, then lack of teacher supportiveness cannot help the bullied child, nor the bully, nor the school climate.

### 4.3. Telling parents about bullying

Some bullied children are afraid to tell a parent who perhaps might urge them to ‘get even’ with the bully. Others might feel ashamed to tell a parent, and fearful that if the parent tells a teacher, and the teacher punishes the bully, then the bully could behave even worse with the bullied child. In this study, 7% of the teens told the younger child to ‘bully back’. If a physically older or stronger peer has bullied a young child, then trying to bully back might just get the bullied child hurt worse. Thus, although the teens were the child's confidantes, the teens did not always have the social/emotional knowledge or more effective practical psychological ‘tools’ that could impact positively to prevent further bullying incidents. Families with older and younger siblings may want to discuss with the teens effective ways to address any bullying confidences given by the younger child in the family to the teen, especially if the parents are aware that a child may worry about disclosing to the parents.

This study did reveal just how reluctant some youngsters may feel about disclosing bullying to parents. Teen responses when asked about whether they had shared the younger child's bullying responses with parents are instructive to consider: *93% of male teens* *did not disclose to parents* what they had learned about the younger child's bullying situation. Also, half of the female teens did not talk with parents about the younger child's disclosures.

When asked about their emotional responses in disclosing the younger child's situation to parents, those teens who had disclosed to parents reported that they felt more negative than positive feelings. Thus, not only younger children but also teens may not feel comfortable about talking with parents about the younger child's bullying situation. This makes the role of teachers and school systems even more important in taking proactive measures designed to curb school bullying episodes.

### 4.4. Schools as sites for mediation and bullying prevention

Schools need to become critical locales for helping students get along more positively. Schools may want to hold sessions to increase teacher awareness of the hurtfulness of *verbal harassment* and *relational forms of bullying*, even when signs are subtle. Some subtle bullying signs that cafeteria monitors may want to watch out for include: children smirking and then moving further apart purposefully on the benches at a school lunch table in order to exclude a child who is coming over to sit at that table, or children whispering and staring with contemptuous grins at a child walking towards them while carrying a tray in the lunchroom.

Often, only physical hurts are addressed as ‘bullying’ episodes in schools. Principals have a responsibility to address a variety of bullying responses in working with teachers. A principal can decide to focus particularly on techniques and measures to address bullying situations while meeting with families in a special school event to address how to improve the social-emotional climate in the school.

High school teachers, counsellors, team coaches and school nurses can be particularly helpful as they engage older youth in discussions with the goal of gaining insights into effective ways to handle any personal bullying situations. School personnel can also facilitate team discussions of ways to assist teens. Group discussions can often produce a wider variety of suggestions, some of which can galvanise teens to help their younger siblings deal more effectively with any bullying encounters in the early grades.

These talks should also include what to do if a child or youth is a *bystander* and sees bullying going on. Student bystander behaviours sometimes goad a bully to escalate and prolong hurtful behaviours. Clear school protocols need to be created for varied school environments, including hallways and lunchrooms, and including the streets just outside of school.

Discussions with teens and with their bullied younger siblings in this study reveal *a need for more proactive school policies*. School policies regarding bullying are not always clear or effective. Sometimes, a three-day expulsion is the only school response to a confirmed bullying episode. Yet, this type of ‘consequence’ does neither heal the negative, scared or rage-filled feelings of the bullied child nor advance the educational goals of the bullying child. Nor will expulsions (without addressing bullying issues on a classroom and on a school-wide basis) help a bully to gain understanding and empathy for others and, just as urgently, also gain insights into possible reasons for the bully's need to denigrate or hurt others. Expulsions alone do not help bullies learn new social skills in how to greet and treat others in the school, whether on the playground, lunchroom or hallways. Group dialogues while seated in a circle with parents and each child present and a social worker or skilled educator as facilitator is one technique that may help a school decrease bullying incidents. Schools and families, working together, can use social skill training techniques to help decrease bullying in schools and increase positive social climates in classrooms and in communities.
